# The Human Papillomavirus (HPV) E6 Oncoprotein Regulates CD40 Expression via the AT-Hook Transcription Factor AKNA

**DOI:** 10.3390/cancers10120521

**Published:** 2018-12-17

**Authors:** Joaquin Manzo-Merino, Alfredo Lagunas-Martínez, Carla O. Contreras-Ochoa, Marcela Lizano, Leonardo J. Castro-Muñoz, Crysele Calderón-Corona, Kirvis Torres-Poveda, Alicia Román-Gonzalez, Rogelio Hernández-Pando, Margarita Bahena-Román, Vicente Madrid-Marina

**Affiliations:** 1 CONACyT-Instituto Nacional de Cancerología, Mexico City 14080, Mexico; 2 Unidad de Investigación Biomédica en Cáncer, Instituto Nacional de Cancerología-Instituto de Investigaciones Biomédicas, Universidad Nacional Autónoma de México, Mexico City 14080, Mexico; lizanosoberon@gmail.com (M.L.); joscasmunoz@gmail.com (L.J.C.-M.); 3 Chronic Infections and Cancer Division, Centro de Investigación Sobre Enfermedades Infecciosas (CISEI), Instituto Nacional de Salud Pública, Secretaría de Salud, Avenida Universidad 655, Col. Santa María Ahuacatitlan, Cuernavaca, Morelos 62100, Mexico; alagunas@insp.mx (A.L.-M.); ccontreras@insp.mx (C.O.C.-O.); crys_calder@hotmail.com (C.C.-C.); kjtorres@insp.mx (K.T.-P.); alycya34@gmail.com (A.R.-G.); mbahena@insp.mx (M.B.-R.); 4 Departamento de Medicina Genómica y Toxicología Ambiental, Instituto de Investigaciones Biomédicas, Universidad Nacional Autónoma de México, Mexico City 04510, Mexico; 5 CONACyT-Instituto Nacional de Salud Pública (INSP), Cuernavaca, Morelos 62100, Mexico; 6 Section of Experimental Pathology, Department of Pathology, Instituto Nacional de Ciencias Médicas y Nutrición Salvador Zubirán, Mexico City 14080, Mexico; rhdezpando@hotmail.com

**Keywords:** HR-HPV, AKNA, E6 oncoprotein, CD40, p53, proteasome

## Abstract

Persistent infection with high-risk Human Papillomavirus (HR-HPV) is the main requisite for cervical cancer development. Normally, HPV is limited to the site of infection and regulates a plethora of cellular elements to avoid the immune surveillance by inducing an anti-inflammatory state, allowing the progress through the viral cycle and the carcinogenic process. Recent findings suggest that the AT-hook transcriptional factor AKNA could play a role in the development of cervical cancer. AKNA is strongly related to the expression of co-stimulatory molecules such CD40/CD40L to achieve an anti-tumoral immune response. To date, there is no evidence demonstrating the effect of the HPV E6 oncoprotein on the AT-hook factor AKNA. In this work, minimal expression of AKNA in cervical carcinoma compared to normal tissue was found. We show the ability of E6 from high-risk HPVs 16 and 18 to interact with and down-regulate AKNA as well as its co-stimulatory molecule CD40 in a proteasome dependent manner. We also found that p53 interacts with AKNA and promotes AKNA expression. Our results indicate that the de-regulation of CD40 and AKNA is induced by the HPV E6 oncoprotein, and this event involves the action of p53 suggesting that the axis E6/p53A/AKNA might play an important role in the de-regulation of the immune system during the carcinogenic process induced by HR-HPV.

## 1. Introduction

The prevalence of different high-risk Human Papillomavirus (HR-HPV) genotypes and their attribution to the development of different types of cancer has been extensively described [1,2,3]. Although most HPV infections are asymptomatic and are cleared by the immune system, there are several factors that favor a small proportion of HPV infections to progress to cervical cancer including HPV genotype, nutritional status and a failure in the immune system [4,5]; nevertheless, the particular contribution of each factor remains under investigation.

Recent findings, suggest that the AT-hook transcriptional factor AKNA, could play a role in the development of CC [6]. This gene is located on chromosome 9q32, a locus of genetic susceptibility to CC [7]. The *akna* gene encodes a transcription factor present in the germinal center of secondary lymphoid organs and immune system cells, such as B and T cells, natural killer and dendritic cells [8]. The N-terminal AT-hook domain of AKNA F1 isoform protein functionally tested in in vitro experiments has shown the ability to bind to the AT-rich promoter regions in both CD40 and CD40 ligand (CD40L), activating their expression to achieve an efficient immune response [8].

CD40 is a transmembrane surface receptor expressed in antigen-presenting cells and other non-hematopoietic cell types including endothelial cells, fibroblasts, smooth muscle cells, and epithelial cells [9,10,11,12,13,14]. The ligand for CD40 is the type II membrane protein CD40L, the CD40–CD40L interaction has a role in both cellular and humoral immune responses [15]. Dendritic cells mature and become active after CD40 ligation, producing high levels of pro-inflammatory cytokines and chemokines [16,17].

Despite all acquired data in the past years, the complete regulatory mechanism for HR-HPV persistent infection remains unknown. Therefore, we hypothesize that CD40 expression, is regulated by the HR-HPV E6 oncoprotein through the transcriptional factor AKNA. In this work, we show the ability of E6 from HR-HPV to bind to AKNA F1 isoform in a series of in vitro and in vivo binding assays. We also demonstrate a minimal expression of this factor and its co-stimulatory molecule CD40 in the HPV-positive cell lines, in comparison with the HPV-negative cell line HaCaT. Furthermore, ablation of E6 expression have a dramatic effect on the recovery of AKNA and CD40 levels, suggesting that AKNA expression is somehow regulated by HR-HPV E6 oncoproteins. These results indicate that the deregulation of AKNA might be a common event in the carcinogenic process induced by the HR-HPVs.

## 2. Results

### 2.1. AKNA Expression Is Ablated in Normal HPV Infected and Neoplastic Cervical Epithelium

**Figure 1:**
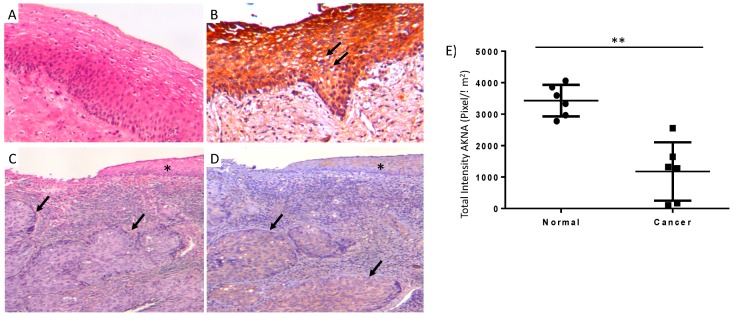
Representative micrographs of conventional histology and immunohistochemistry to detect AKNA in normal and neoplastic cervical epithelium. (**A**) Normal histological appearance of uterine cervix, showing squamous stratified epithelium supported by connective tissue. (**B**) Normal epithelium showing strong AKNA immunostaining. Arrows indicate koilocytes where AKNA staining is absent. (**C**) Intense cervicitis with small fragment of epithelium (asterisk) and large nodules of neoplastic cells in subjacent connective tissue that correspond to infiltrating areas of mild differentiated squamous cell carcinoma (arrows). (**D**) Conserved epithelium (asterisk) and neoplastic nodules (arrows) show slight AKNA immunostaining (panels **A** and **B** 200× magnification, panels **C** and **D** 100× magnification). (**E**) Digital pathology study confirmed AKNA reduced levels in cancer cases (*n* = 6) compared to normal epithelia (*n* = 6) (** *p* < 0.01).

A recent study demonstrated that the transcription factor AKNA is associated to an increased risk for developing cervical cancer [6]. We evaluated the production of AKNA in cervical biopsies from normal epithelium, cervicitis and infiltrating squamous cell carcinoma in 12 cases of hysterectomy. The results demonstrate a strong AKNA immunostaining in areas of normal cervical epithelium (Figure 1A,B) that progressively decrease in areas of cervicitis, being almost negative in areas of well and moderate differentiated squamous cell carcinoma (Figure 1C,D). Also, we analyzed 6 cases of cervical epithelium with morphological changes induced by HPV infection (koilocytes) where AKNA staining was completely negative in the koilocytes (Figure 1B, arrows). Additionally, we quantified the AKNA staining in the analyzed cases finding significantly lower protein levels in cancer cases compared to normal epithelium (Figure 1E); although the cancer cases showed a wide range of AKNA signal indicating the tumor heterogeneity, the difference was clear when comparing with normal tissue. Thus, normal cervical epithelium shows strong AKNA production while in areas of dysplasia and local or invasive squamous cell carcinoma, there is a substantial decrease of AKNA production.

### 2.2. AKNA is Down Regulated in HPV Positive Cell Lines

**Figure 2:**
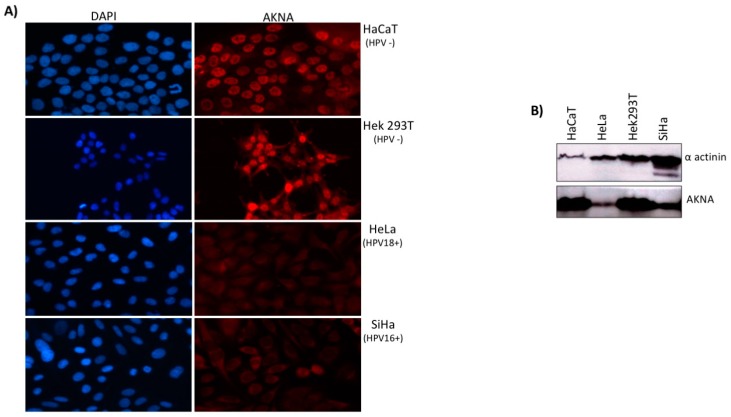
AKNA is down-regulated in HPV positive cells. (**A**) HaCaT, HEK293T, HeLa and SiHa cells were seeded on a coverslip in a 6 well plate and incubated to allow adherence, 24 h after seeding cells were fixed with PBS/3.7% para-formaldehyde for 10 min, rinsed and incubated with AKNA anti-serum (this study) followed by Alexa 555 coupled secondary antibody (Molecular probes). After several washes, the slides were mounted in Prolong DAPI media (Molecular probes) and analyzed in a fluorescence microscope EVOS FL (Thermo Fisher Scientific, Waltham, MA, USA). The HPV positive cell lines showed a weak staining in the nucleus (HeLa) and cytoplasm (SiHa) for AKNA compared to the HPV negative cell lines HaCaT and HEK293T. All images were acquired under a 40× objective with the EVOS system. (**B**) AKNA protein levels were analyzed by western blot in different cell lines using the AKNA anti-serum. α-actinin was used as a loading control. HPV positive cells (HeLa and SiHa) exhibited a lower AKNA protein levels.

Since deregulation of the immune system by HPV determines persistence of the infection and is highly implicated in cancer progression, we wonder whether the HPV might be regulating AKNA. We examined AKNA levels in different HPV-positive cell lines including SiHa (positive for HPV16) and HeLa (positive for HPV18) as well as two HPV-negative cell lines HaCaT and HEK293T, using an immunofluorescence approach and by western blot. AKNA immunostaining was detected at a higher level in the HPV-negative cell lines compared with those HPV-positive (Figure 2A).

HEK293T and HaCaT cells exhibited a nuclear staining (Figure 2A upper panels), whilst HeLa and SiHa cells show a very weak AKNA signal, which interestingly, it was mainly detected at the nucleus and cytoplasm (Figure 2A bottom panels). The western blot analysis demonstrated lower levels of AKNA protein in the HPV positive cells lines HeLa and SiHa (Figure 2B), suggesting that HPV oncoproteins might be associated to AKNA downregulation.

### 2.3. HR-HPV E6 Oncoproteins Decrease AKNA Protein Levels Involving the Proteasome System

**Figure 3:**
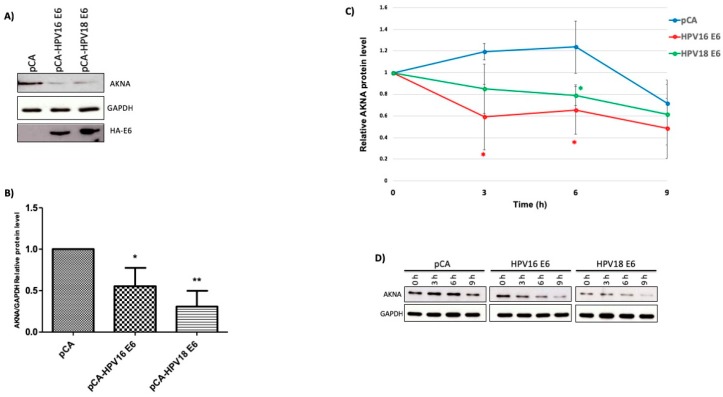

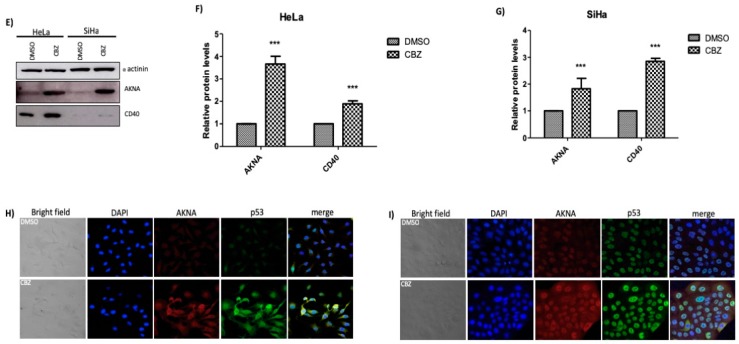
HPV E6 oncoproteins regulate AKNA levels. (**A**) HaCaT cells were transfected with plasmids expressing HA-tagged versions of HPV16 and 18 E6 oncoproteins, 24 h after transfection, cells were collected, and protein extracted by adding sample buffer. The levels of AKNA were ascertained by western blot using AKNA anti-serum. Anti-HA antibody was used to determine the expression of the HPV E6 oncoproteins. E6 expressing cells show a clear reduction in AKNA levels 24 h post-transfection compared with the control vector (pCA). These results demonstrate that E6 is able to regulate AKNA protein levels. (**B**) Densitometric analysis expressing data as the ratio of relative units between AKNA and GAPDH. Data are shown as the mean ± SD. Tukey’s test * *p* < 0.05 and ** *p* < 0.005 vs. pCA control. *n* = 3. (**C**) After Cycloheximide treatment, protein extracted after 0, 3, 6 and 9 h of treatment and analyzed for AKNA levels. The graph presents data as the mean ± SD. Tukey’s test * *p* < 0.05 vs. pCA control. *n* = 3. AKNA levels were diminished in E6 expressing cells. (**D**) Representative immunoblot showing the different treatments. (**E**–**I**) HeLa, SiHa and HaCaT cells were seeded on coverslips in 6 well plates. 24 h after seeding, cells were treated either with proteasome inhibitor (CBZ) or DMSO (vehicle) for 3. After the incubation time, cells were either collected and protein extracted and analyzed or fixed and stained using AKNA anti-serum followed by Alexa 488 (green) or Alexa 555 (red) secondary antibodies (Molecular Probes). (**E**) Western blot results showing an increase in AKNA levels after proteasome inhibition. α-actinin was evaluated as loading control. (**F**,**G**) Densitometric analysis expressing data as the ratio of relative units between AKNA and CD40 over α-actinin in HeLa and SiHa cells, respectively. Data are shown as the mean ± SD. Student’s *t*-test *** *p* < 0.001 vs. DMSO treated cells (control). *n* = 3. Stained slides were mounted using Prolong DAPI media (Molecular probes) and observed under the confocal NIKON Eclipse Ti microscope. (**H**) HPV positive cell line HeLa exhibited a recovery in the levels of p53 in the nucleus as expected and AKNA levels were also recovered after proteasome inhibition mainly in the nucleus. (**I**) HaCaT cells showed a higher signal of AKNA and an accumulation after proteasome inhibition. All images were acquired under the 40× objective using a NIKON camera.

To determine whether AKNA could be a target of the HR-HPV E6 oncoproteins, we transfected HaCaT cells with plasmids expressing HA-tagged versions of HPV16 and HPV18 E6. After 48 h, cells were harvested, and protein analyzed by SDS-PAGE and western blot. As shown in Figure 3A,B, E6 proteins have the ability to decrease AKNA levels when compared with the control cells transfected with the empty vector (pCA). Additionally, a half-life assay was performed in HaCaT cells transfected with E6 expressing plasmids. AKNA levels were decreased at 3 and 6 h after cycloheximide treatment in HPV18 E6 expressing cells, whilst AKNA levels decreased at 6 h after treatment in HPV16 transfected cells. AKNA levels were comparable low in cells harboring E6 either from HPV16 or 18 (Figure 3C,D) when comparing with the control transfected cells, where AKNA levels decreased at 9 h. GAPDH showed equal amounts of loaded protein. We also evaluated p53 which levels were reduced at 3 h in control cells (pCA) as expected. Whilst E6 expressing cells showed reduced levels from time 0, due to the action of the oncoproteins. These results demonstrate the ability of HR-HPV E6 to induce a reduction in AKNA levels.

HR-HPV E6 oncoproteins have the capacity to bind to the E3 ubiquitin-protein ligase E6AP inducing the ubiquitination of many cellular proteins leading ultimately to their degradation, which represents one of the main processes by which the HPV E6 regulates cellular protein levels via the proteasome [18].

To probe whether the proteasome is involved in the low levels of AKNA protein exhibited by HPV-positive cell lines, we analyzed AKNA levels in HeLa, SiHa and HaCaT cells, by western blot and immunofluorescence, using proteasome inhibitors (Z-Leu-Leu-Al). The western blot analysis demonstrated a significant increase in AKNA levels after proteasome inhibition in the HPV-positive cell lines HeLa and SiHa, indicating that AKNA is regulated by the proteasome in those cell lines (Figure 3E–G). Interestingly, CD40 protein levels were also upregulated after proteasome inhibition, probably as an AKNA-related effect (Figure 3F,G). To further analyze the recovering of AKNA after proteasome inhibition, we performed an immunofluorescence analysis staining AKNA; the p53 stain was used as a positive control for proteasome inhibition. Figure 3H shows p53 positive stain in the nucleus after proteasome inhibition in HeLa cells, as expected. AKNA signal was also recovered after the treatment, exhibiting nuclear and cytoplasmic distribution (Figure 3H). In contrast, when analyzing HaCaT cells, high levels of AKNA staining were detected mainly in the nucleus (Figure 3I) with a massive accumulation after proteasome inhibition, suggesting that AKNA could be transcriptionally activated in these cells. To probe the specificity of the AKNA antibody, we tested the levels of AKNA using a commercially available antibody (Abcam, Cambridge, UK) aimed to detect the F1 isoform of AKNA. After proteasome inhibition, HeLa and SiHa cells exhibited the recovery of AKNA signal mainly in the nucleus in a spotted pattern (Supplementary Figure S1A,B). C33A cell line was included as an HPV negative control for this experiment, surprisingly, we did not detect any increase in AKNA signal after proteasome inhibition and basal levels were also low, as those shown in HPV positive cell lines (Supplementary Figure S1C).

These results demonstrate not only that the proteasome has a significant role in AKNA regulation in HPV-positive cell lines, but also raises the question whether p53 might play a role in regulating AKNA in the absence of HPV proteins, since the p53 mutant C33A cell line exhibits an absent signal for AKNA and no recovery was found after proteasome inhibition.

### 2.4. HPV E6 Oncoproteins and p53 Interact with AKNA In Vivo and In Vitro

**Figure 4:**
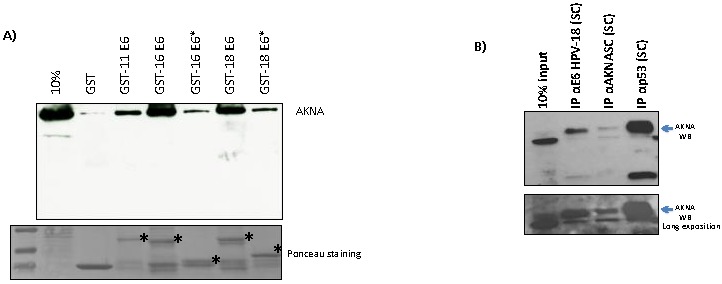
HPV E6 oncoproteins interact with the AT-hook factor AKNA. (**A**) Purified GST-E6 fusion proteins were incubated for 4 h with a HaCaT cell lysate to allow complex formation. After incubation, GST beads were washed using 0.1% NP-40/PBS solution to wash away unspecific binding. Bound protein was analyzed by western blot using AKNA anti-serum. GST alone was incubated as a binding control. AKNA protein is detected when incubated with GST-E6 proteins. GST-E6 fusion proteins are indicated by the asterisk (*). (**B**) Anti-E6 (1:25) directed against HPV18 (Santa Cruz Biotechnologies) was used to immunoprecipitate endogenous E6 in HeLa cells, after CBZ treatment to ensure high levels of protein. Anti-AKNA (1:50, Santa Cruz Biotechnologies) and anti-p53 DOI (1:100, Santa Cruz Biotechnologies) and anti-E6 (HPV18) antibodies were incubated overnight with a HeLa cell lysate obtained using RIPA buffer, after that time IgG beads were added to the cell lysate and incubated for 2 h. After several washes bound protein was ascertained by western blot using AKNA anti-serum. Western blot analysis revealed that AKNA is able to complex with E6. p53 was used as an immunoprecipitation control. Longer exposure (lower panel) is showed to demonstrate the presence of AKNA in the input. Note the appearance of AKNA bands detected when p53, AKNA and E6 are immunoprecipitated indicating the in vivo interaction between the analyzed proteins.

It is well known that E6 regulates a plethora of cellular proteins due to its ability to interact with them [19]. To test the capacity of E6 to bind to AKNA we perform a GST-pull down assay using GST-E6 fusion proteins from different HPVs. Figure 4A shows a clear interaction between the different E6 proteins and AKNA whilst E6* isoforms showed weak interaction, GST balance is shown in the bottom panel to demonstrate the presence of the GST fusion proteins. The low risk HPV E6 protein also showed the ability to interact with AKNA indicating that this is a shared feature among the HPVs. To further explore the E6 ability to interact with AKNA we perform a series of immunoprecipitation assays with anti-AKNA, anti-p53 and anti-E6 antibodies, aimed to precipitate endogenous HPV18 E6 in HeLa cells. Figure 4B shows that the E6 protein precipitated from HeLa cells was able to interact with AKNA. We performed a specificity binding control using anti-p53 antibody discovering that AKNA protein was also detected when p53 was immunoprecipitated, indicating a probable trimer complex composed by E6, p53 and AKNA since AKNA is also interacting with E6. AKNA was also immunoprecipitated using anti-AKNA (Santa Cruz Biotechnologies, Dallas, TX, USA) as a confirmatory control for the presence of the AKNA protein.

### 2.5. HPV E6 Regulates the Levels of AKNA and CD40

**Figure 5:**
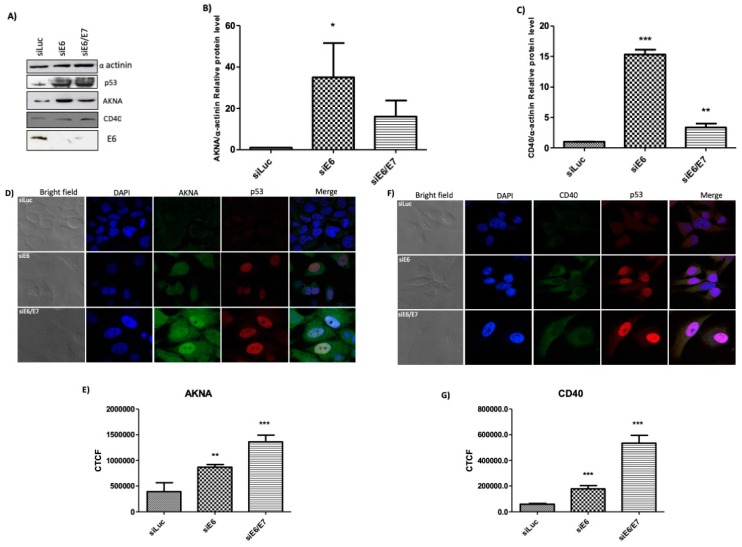
E6 regulates the expression levels of AKNA and CD40 in the HPV positive cell line HeLa. HeLa cells were seeded in a 60 mm dishes and transfected with siRNAs directed against E6, siE6/E7 and Luciferase (siLuc) as an unspecific control. After 72 h cells were either fixed or collected and total protein extracted using sample buffer. (**A**) Levels of AKNA and CD40 were evaluated by western blot using specific antibodies, α-actinin was used as a loading control, and p53 was evaluated to assess the silencing efficiency of E6. Cells showed a clear increase in p53 levels indicating the efficient ablation of E6 expression and, in cells were p53 was restored showed also an increase in AKNA and CD40 levels. Densitometric analysis expressing data as the ratio of relative units between AKNA/α-actinin (**B**), and CD40/α-actinin (**C**). Data are shown as the mean ± SD. Tukey’s test * *p* < 0.05, ** *p* < 0.001 and *** *p* < 0.0001 vs. pCA control. *n* = 3. (**D**,**F**) Fixed HeLa cells were stained for immunofluorescence analysis using anti-CD40, AKNA anti-serum and anti-p53 antibody followed by secondary Alexafluor antibodies as specified, finally cells were mounted using Prolong DAPI media (Molecular Probes) and observed in a confocal NIKON Eclipse Ti microscope. E6 knocked down cells exhibited p53 positive signal due to E6 inactivation, p53 positive cells showed a clear accumulation of AKNA in the cytoplasm and in the nucleus as well as a co-localization with p53. CD40 showed an increase in the siE6 and siE6/E7 cells mainly in the cytoplasm. All images were acquired under the 40× objective using a NIKON camera. (**E**,**G**) AKNA and CD40 fluorescence quantification using the corrected total cell fluorescence (CTCF) method. Data are shown as the mean ± SD. Student’s *t*-test ** *p* < 0.01 and *** *p* < 0.0001 vs. siLuc control. *n* = 3.

The ability of HR-HPV E6 to induce degradation of its cellular partners has been extensively studied and numerous cellular models have been established so far. Among these, knocking down E6 expression in HeLa cells has shown to be a reliable and comparable model to study the influence of E6 on its cellular targets [20]. To demonstrate whether E6 is involved in the regulation of AKNA levels, we knocked down E6 expression in HeLa cells by using siRNAs. HeLa cells were transfected with siRNA against E6 and E6/E7. A siRNA directed against Luciferase was used as an unspecific control. After 72 h of incubation, attached cells (viable cells) were either harvested and protein obtained, and analyzed by western blot or immunostained for immunofluorescence analysis. Western blot results (Figure 5A) demonstrate a high recovery in p53 levels after silencing E6 and E6/E7 as expected. AKNA levels as well as the co-stimulatory molecule CD40 were also recovered in the same conditions compared with the siLuciferase control, showing that its expression depends on AKNA recovery (Figure 5A–C). 

To examine the localization of the recovered proteins, an immunofluorescence analysis was performed. Figure 5D, shows that after E6 or E6/E7 knocking down p53 signal increase in the nucleus as previously reported [21,22] backing up the efficacy of E6 ablation. Following E6 silencing, AKNA stain becomes stronger mainly in the nucleus and CD40 signal was located mainly in the cytoplasm (Figure 5D,F). Confocal analysis also revealed a nuclear localization of p53 and AKNA, indicating that both proteins might be interacting at that specific location, hence supporting the immunoprecipitation results (Figure 4B). Fluorescence quantification confirmed the increase of AKNA (Figure 5E) and CD40 (Figure 5G) after E6 or E6/E7 silencing. Taken together these results demonstrate that E6 is regulating AKNA and subsequently CD40 levels in vivo.

### 2.6. Restoration of p53 Increases the Levels of AKNA and CD40 in HPV-Positive Cell Lines

The immunoprecipitation assays showed that p53 interacts with AKNA. Also, the rescue in CD40 levels after AKNA recovery when E6 expression is ablated, raised the inquiry whether p53 might be regulating AKNA expression. Additionally, the fact that the p53 mutant cell line C33A does not show any recovery in AKNA levels after proteasome inhibition suggest a role for p53 in regulating AKNA levels. To demonstrate if p53 could promote AKNA and CD40 expression we transfected HeLa cells with a p53 expressing plasmid and evaluated the protein levels 24 h after transfection by western blot and by immunofluorescence. 

**Figure 6:**
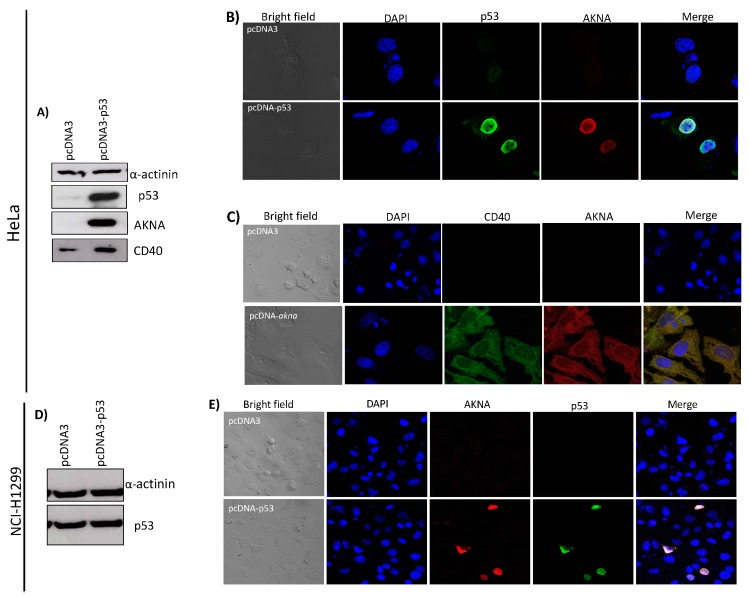
AKNA is expressed after p53 restoration in HeLa and NCI-H1299 cells. (**A**) HeLa cells were transfected with a p53 expressing plasmid (pcDNA3-p53). After 24 h of transfection, cells were harvested, and total protein extracted using sample buffer. AKNA, CD40 and p53 protein levels were ascertained by western blot using AKNA anti-serum, anti-CD40 and anti-p53 antibodies. As expected p53 protein levels increased in cells transfected with the pcDNA3-p53 plasmid and AKNA levels become visible as well as CD40 protein levels in the same cells compared with the cells transfected with the control vector (pcDNA3). α-actinin was used as a loading control. (**B**) HeLa cells were transfected, fixed and stained for immunofluorescence analysis using AKNA anti-serum, anti-p53 and anti-CD40 followed by Alexa 488 and 555 secondary antibodies. p53 expressing cells showed a massive recovery in AKNA signal in the nucleus indicating the p53 dependent effect of AKNA. (**C**) HeLa cells were transfected with either pcDNA3 or the pcDNA3-*akna* plasmid, then cells were fixed and stained for immunofluorescence analysis using AKNA anti-serum and anti-CD40 antibodies followed by Alexa 488 and 555 secondary antibodies. Cells expressing AKNA exhibit a strong CD40 signal. (**D**) NCI-H1299 cells were transfected with pcDNA3-p53 plasmid. Twenty-four h after transfection, cells were collected and p53 expression assessed by western blot. (**E**) NCI-H1299 transfected cells were fixed and stained for immunofluorescence analysis using AKNA anti-serum and anti-p53 antibodies (Santa Cruz Biotechnology) followed by Alexa 488 and 555 secondary antibodies. p53 expressing cells show an evident AKNA signal. Slides were mounted using Prolong DAPI media (Molecular probes) and observed in a confocal NIKON Eclipse Ti microscope. All images were acquired under the 40× objective using a NIKON camera.

Results in Figure 6 clearly show that AKNA expression is induced after p53 is over-expressed with the subsequent rescue of CD40 protein when we analyzed the protein levels by western blot (Figure 6A), compared with the cells transfected with the control vector. The immunofluorescence analysis (Figure 6B) probed that p53 was expressed in the nucleus and that those p53 expressing cells have a massive recovery of AKNA levels mainly at the nucleus as previously determined (Figure 5). Additionally, we transfected HeLa cells with an AKNA expressing plasmid finding that CD40 was recovered in the cytoplasm when AKNA is expressed (Figure 6C).

To further analyze the effect of p53 on AKNA levels, we used the NCI-H1299 cell line which lacks the expression of p53 [23]. NCI-H1299 cells were seeded and transfected with a p53 expressing plasmid (pcDNA3-p53). After 24 h, cells were either collected and total protein extracted or fixed and processed for immunofluorescence analysis. Immunoblot shows a successful expression of p53 (Figure 6D) The immunofluorescence analysis showed that p53 is expressed in the nucleus of the NCI-H1299 transfected cells and AKNA is also expressed in p53 positive cells, indicating a direct role for p53 in the regulation of AKNA expression (Figure 6E). These results point out AKNA as a new cellular target for p53 and indicate that HPV E6 oncoprotein could modulate AKNA functions in an indirect manner due to p53 negative regulation in HPV positive cell lines.

### 2.7. CD40 AKNA-Induced Expression in HeLa Cells Does not Localizes at the Cell Membrane

CD40 levels are increased either by ablating E6 (Figure 5) or by overexpressing AKNA (Figure 6). Nevertheless, CD40 requires being located at the cell membrane in order to promote the inflammatory phenotype. We tested whether CD40 might be expressed on the surface of HeLa cells after AKNA restoration. Flow cytometry analysis showed no significant differences on CD40 extracellular expression in HeLa cells transfected with empty vector pcDNA3 (15.4%) or pcDNA3-*akna* (19.2%). Indicating that the CD40 molecules require an additional stimulus in order to be translocated to the membrane, at least in the conditions tested in this study.

### 2.8. Restoration of AKNA Levels in HeLa Cells Induces the Expression of Interleukin 8 (IL-8)

**Figure 7:**
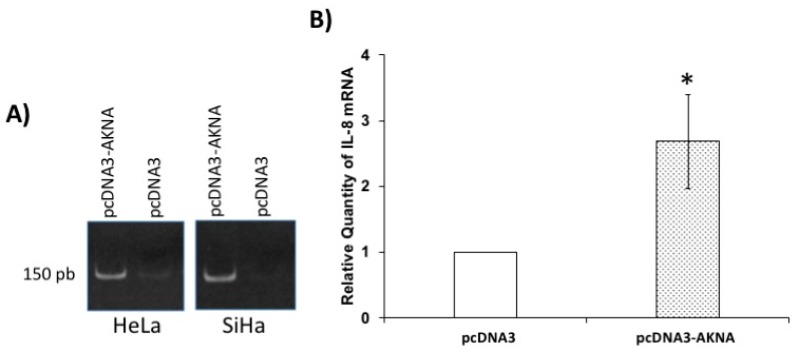
AKNA restoration induces expression of IL-8 in HPV positive cell lines. HeLa cells were transfected with pcDNA3-*akna* plasmid and after 24 h, total RNA was extracted using Trizol following the manufacturer’s instructions. Complementary DNA was synthesized and used as a template for IL-8 mRNA amplification using standard PCR (**A**) or relative quantification (**B**). AKNA transfected cells showed a remarkable increased in the IL-8 mRNA levels after AKNA expression. Data is presented as relative expression of IL-8 after normalizing with the HPRT1 expression ± SD. * *p* = 0.017 vs. pcDNA3 control. *n* = 4.

We have demonstrated an evident effect of E6 on AKNA and CD40 levels. Normally, AKNA regulates the expression of CD40 and CD40L [8]. After CD40 activation the expression of co-stimulatory molecules is induced, including interleukin 8, promoting a pro-inflammatory state in order to recruit the modulators of the immune response at a local level [24,25]. Since E6 modulates AKNA and CD40 levels and one of the targets for CD40 is interleukin 8, we wondered whether the restoration of AKNA induced IL-8 expression. To test this, we transfected HeLa cells with an AKNA expressing plasmid and evaluated IL-8 mRNA expression by qPCR. Figure 7A shows the appearance of a 150 base pairs product corresponding to IL-8 mRNA in AKNA-expressing cells HeLa and SiHa, in comparison with those with the control vector at 24 h after transfection. This effect was consistent either in HeLa and SiHa cells at 48 h after transfection. Quantitative PCR showed statistically significant IL-8 higher levels when AKNA was present in HeLa cells; expression was used as a normalizing control (Figure 7B). The increase in IL-8 mRNA demonstrates a functional effect for AKNA in HeLa cells and supports the idea that inflammatory status is avoided by the HPV via p53/AKNA modulation.

## 3. Discussion

Lacking of AKNA expression has been associated with an increased risk for CC development [6]. In this study, we identified AKNA as a novel target of degradation for the HPV E6 oncoprotein involving the proteasome system. We also demonstrated that CD40 expression is altered in the HPV positive cell line HeLa and that ablation of E6 induces a recovery in AKNA and CD40 levels. One consequence of the restoration of AKNA expression in HeLa cells was the increased expression of interleukin 8 which might indicate a negative regulation of the pro-inflammatory signals involved in the elimination of HPV transformed cells. Moreover, in good correspondence with these results, cervical normal epithelium shows strong AKNA immunostaining, while malignant neoplastic cervical epithelium, as well as dysplastic cervical epithelium with morphological changes induced by HPV infection (koilocytes) were completely AKNA negative.

The HPV E6 oncoproteins regulate a plethora of cellular functions by inducing the degradation of cellular proteins, due to the ability of HPV E6 to interact with them in a protein-protein fashion. Although AKNA has not been reported as an interacting partner for HPV E6 oncoproteins [26,27], we were interested in testing such ability through IP and Pull down experiments to demonstrate protein-protein interaction. Indeed, we showed that HPV E6 oncoprotein is capable of interacting with AKNA, specifically with the F1 isoform (upper band), which has been tested as the biologically active form of AKNA [28]. The IP analysis revealed that E6 as well as p53 interact with this isoform, although another smaller band was detected probably indicating that another isoform harboring a common region with the AKNA F1 is capable to form a complex with both E6 and p53. Interestingly, the capacity of E6 to interact with AKNA is shared with p53, since when p53 is immunoprecipitated we found AKNA as part of the complex and the immunofluorescence analysis shows a clear co-localization between p53 and AKNA in the nucleus after E6 ablation, indicating a probable trimer complex involving E6-p53-AKNA; nevertheless, further analyses are required to demonstrate the regulatory effects of this complex. In this regard, a recent study indicated that the PKA/CREB and NFκB/p65 pathways negatively regulate AKNA, inducing a down-regulation in the levels of inflammatory citokines [29]. Our results suggest an important role for p53 in the regulation of AKNA levels as demonstrated by the increment in the levels of AKNA after promoting p53 expression, whether this regulation requires the p53-transcriptional functions or a p53-induced protein stabilization, as well as the cellular processes affected by this interaction requires further studies.

The fact that p53 is found when AKNA is precipitated leads to the question of whether E6 regulates the AKNA-p53 complex, and in consequence the effect observed for AKNA in this study might be merely a p53 degradation side effect, or what are the cellular effects induced by this novel complex. Still, it is necessary to further investigate these concerns.

HPV E6 associates to the E3 ubiquitin ligase E6AP and consequently employs the 26S proteasome system to promote degradation of its cellular partners [30]. Our results probe that the proteasome system is implicated in AKNA deregulation but the participation of E6AP in this process remains to be explored. It would be important to test whether AKNA is recognized and subsequently degraded by the E6/E6AP complex. If so, the study of the possible consequences will provide the basis of the E6/p53/AKNA complex in the oncogenic process. Nonetheless, the protein levels of AKNA and CD40 were recovered after proteasome inhibition indicating the involvement of the proteasome in the regulation of AKNA and its activities. The proteasome inhibition showed an effect in AKNA recovery in HPV positive cells as shown in the western blot, this fact is further supported by the immunofluorescence analysis as well as by the E6 ablation, demonstrating a dramatic effect in AKNA and CD40 protein rescue. Whether AKNA could be degraded as a consequence of p53 degradation induced by the E6 oncoprotein, remains an open question. Nevertheless, it is well known that E6 oncoproteins decrease p53 half-life [31]. We demonstrated that AKNA levels are decreased by the E6 oncoproteins at 3 and 6 h, which normally is about 9 h. Additionally, our immunofluorescence assays show clearly that AKNA has both cytoplasmic and nuclear distribution. Thus, there is some AKNA uncomplexed that is probably downregulated by E6 in an p53-independent manner.

CD40 is overexpressed in several carcinomas including cervical, ovarian and gastric cancers where its function remains largely unknown [32]. In HeLa cells CD40 expression is contradictory, was positive by western blot [33], but on cell surface was negative by FACS [34,35]. However, CD40 was significantly higher in cervical carcinoma than in normal cervix, maybe involved in neovascularization or enhanced by a cytotoxic immune response [35,36]. The fact that CD40 is recovered either by proteasome inhibition or E6 ablation in HeLa cells, indicates a possible role for CD40 in the modulation of immune surveillance and represents basic findings for the study of CD40 novel actions in the development of HPV derived cancers. Even though the role of CD40 has been widely demonstrated in cells of the immune system [16], Borcherding and co-workers have demonstrated that epithelial cells exhibit a remarkable expression of CD40 during chronic inflammation [37], mainly in the cytoplasm, indicating possible different roles for CD40 regarding its cellular localization. Regarding CD40 expression in cervical cancer tissues, some reports indicate that CD40 signal becomes stronger in cancer compared to low-grade or normal epithelium [35]. Nevertheless, other studies indicate that not all cervical cancer cases display CD40 signal [36] or even, most of the cancer cases analyzed exhibit a weak signal for CD40 staining [38]. In cases where CD40 is absent it is possible that HPV proteins could play an important role in downregulating CD40 levels promoting the advance of cervical lesions.

Although the CD40 levels seem to be lower by western blot than by IF, the statistical analysis indicates that the recovered amount of CD40 is significantly higher when E6 is knocked down probably due to the differences in the techniques used. Importantly, we did not find CD40 at the cell surface after AKNA expression in HeLa cells, instead CD40 shows a cytoplasmic accumulation either after silencing E6 or expressing AKNA. Furthermore, the upregulation in IL-8 after AKNA indicates that CD40 is somehow involved in the transcriptional effect. However, whether AKNA or cytoplasmic CD40 is the responsible for the up-regulation in IL-8 remains to be explored, not only to know what other molecules are regulated by AKNA, but CD40 raises as a potential regulatory molecule for inflammatory cytokines since it has been demonstrated that nuclear CD40 is capable of interacting with the transcriptional activator c-Rel promoting inflammatory related genes [39].

The fact that IL-8 mRNA is stimulated even in the absence of the classic membrane localization of CD40 raise an important issue regarding the biological actions of CD40 mediated signaling. In this regard, there is cumulative evidence indicating that endosome-contained receptors have the capacity to promote different cellular responses by signaling independently of the membrane location, due to the action of endosomal scaffold proteins [40,41,42,43]. Additionally, it has been demonstrated that internalized CD40 activates the NF-κB pathway [44]. The authors suggest that CD40 forms an intracellular signaling complex, which could be supported by our results where CD40 is located at the cytoplasm after being recovered by E6 ablation. Whether intracellular CD40 is indeed signaling remains to be explored.

It is well known that HPV infection courses without an inflammatory phenotype although at the end in most of the cases, the immune system clears the infection. Nevertheless, in some cases HPV exerts different mechanisms to avoid immune recognition and elimination [45,46,47]. A genome-wide expression profile demonstrated that human keratinocytes expressing episomal HPV genomes induced the downregulation of pro-inflammatory cytokines and chemokines suppressing immune recognition [48]. Additionally, it has been demonstrated that HPVs suppress the innate immune response in keratinocytes due to the ablation of cytokines and chemokines production, which normally control the attraction and activation of an adaptive immune response in epithelial cells [49]. Also, the HPV has the ability to impair the CD40-mediated local immunity [50], suggesting that the HPV attenuated response in epithelial cells might have a role in the viral elimination and further persistency. Interestingly, the study of human samples showed strong AKNA immunostaining in inflammatory cells around the neoplastic epithelium which was AKNA-negative or slightly positive, suggesting an inherent AKNA deficiency in neoplastic epithelial cells that is not exhibited by immune cells that conform the inflammatory infiltrate related to the cancerous epithelium.

These results further support the idea that HPVs deregulate the inflammasome networks, and our results suggest that the deregulation of AKNA by HPV E6 oncoprotein might participate in this process. Moreover, to decipher the altered processes by the different viral proteins remains as an important issue for the understanding in the HPV induced carcinogenesis.

## 4. Materials and Methods

### 4.1. Histology and Immunohistochemistry Studies in Human Tissue

The cervical tissue from 10 cases of hysterectomy for squamous cell carcinoma as well as 4 cases of normal cervical epithelium were obtained from the Pathology Department of the National Cancer Institute from Mexico, Mexico City, after the corresponding approvals of the Ethical and Research Committees references: (CEI/1284/18) (018/037/IBI). Resected uterus was conventionally sectioned and studied as part of the histopathological diagnosis. Areas of normal epithelium, cervicitis with dysplastic epithelium and local and infiltrative squamous cell carcinoma were selected for conventional histology and immunohistochemistry using the same paraffin blocks for both studies.

Paraffin imbedded tissue was sectioned 2 μm tick and mounted on glass slides, then deparaffinized and processed either for hematoxilin and eosin staining or for AKNA-IHC. IHC slides were rehydrated through ethanol, then epitope retrieval was done using a pressure cooker using the ImmunoRetriever Citrate Solution (Bio SB, Santa Barbara, CA, USA) during 12 min. After cooling down the slides the tissues were treated for 1 h with the PolyDetector Peroxidase Block quenching buffer (Bio SB). Non-specific binding for the antibody was blocked using 10% BSA solution (30 min). Slides were incubated with mouse anti-AKNA serum (this study) at a dilution of 1:50 for 2 h at room temperature. Following two washes in PBS, slides were incubated for 30 min with a secondary antibody (PolyDetector HRP label; Bio SB). After three washes in PBS, sections were incubated with the appropriate substrate (PolyDetector DAB chromogen; Bio SB), counterstained with hematoxylin and mounted in Entellan New mounting reagent (Merck, Kenilworth, NJ, USA).

### 4.2. Digital Imaging and Analysis of Immunohistochemical Staining

AKNA stained sections were scanned and digitalized using the Aperio Scanscope CS obtaining a digital 40× images. Then, sections were annotated and AKNA staining was quantified with the Spectrum software (Aperio/Leica Microsystems, Wetzlar, Germany) according to Rodriguez et al., 2015 [51]. The quantitative intensity of AKNA signal was graphed into two groups: normal cervix and cancer, and statistical analysis was performed.

### 4.3. Cell Culture and Transfection

HeLa, SiHa, NCI-H1299 and C33-A cells were obtained for the American Typing Culture collection (ATCC, Manassas, VA, USA) and maintained in Eagle’s Minimum Essential Medium, U-2 OS cells were maintained in McCoy’s 5a medium, HaCaT cells were kind gift from Dr. Alejandro García-Carrancá (Instituto Nacional de Cancerología, México, México) and were maintained in DMEM-F12 medium. Primary human keratinocytes were cultured in DMEM/F12 media supplemented with grown factors [52]. All media was supplemented with 10% fetal bovine serum (FBS). Transfections were performed using Polyfect reagent (Qiagen, Hilden, Germany) or Lipofectimine 2000 (Invitrogen, Carlsbad, CA, USA) according to the manufacturer’s instructions. For silencing experiments, HeLa cells were seeded in a 6-well plate at a confluence of 1.2 × 10^5^ and transfected using Lipofectamine RNAiMAX reagent (Invitrogen) with siRNAs directed against Luciferase, HPV18 E6/E7 (5′-CAUUUACCAGCCCGACGAG) or HPV18 E6 (5′-CUCUGUGUAUGGAGACA CATT) acquired from Thermo Scientific Dharmacon (Lafayette, CO, USA), 72 h after transfection, cells were washed and processed for either western blot or immunofluorescence analysis.

### 4.4. Plasmids

pcDNA3-AKNA and pcDNA-p53 were generated by cloning the coding region using standard PCR techniques. GST-E6 fusion plasmids were generated by cloning the different open reading frames of E6 into the pGEX-2T vector (GE, Chicago, IL, USA), as previously reported [21]. HA-tagged E6 expressing plasmids were generated by cloning the E6 coding regions into the pCA vector using standard PCR techniques as previously reported [21].

For AKNA expressing plasmid generation, peripheral blood mononuclear cells (PBMC) were isolated by Ficoll Hypaque gradient (Sigma Aldrich, St. Louis, MO, USA). Total RNA was extracted from PBMC using Trizol (Sigma Aldrich) reagent according to the manufacturer’s recommendations and cDNA was generated. Then, the AKNA F1 isoform was amplified by PCR using the primers AKNA-Fwd 5′-GGAATTCAGACTACCACAGGCTCCT and AKNA-Rev 5′-GCTCTAGAGTGAAGTCAG AAGAGGCA, which included the EcoRI and XbaI restriction sites, respectively. The PCR product was cloned into the pCRII-TOPO vector (Thermo Fisher Scientific, Waltham, MA, USA). After, we sub-cloned this product into the pcDNA3 vector (Invitrogen) using the sites previously mentioned. All plasmids were verified by sequencing.

### 4.5. Western Blot

Cells were transfected with the indicated plasmids and 24 h after transfection the cells were washed and either lysed directly with sample loading buffer or with RIPA buffer to extract protein. Cell extracts were analyzed by SDS-PAGE and blotted onto a 0.22 μm nitrocellulose membrane (GE). Membranes were blocked with 7.5% milk/0.1% Tween-20/TBS at 37 °C for 30 min, washed tree times 10 min each with TBS/0.1% Tween-20 and then incubated with primary antibodies diluted in TBS/0.1% Tween-20/3.5% milk for 2 h at room temperature or at 4 °C overnight with continuous agitation. After primary incubation, membranes were washed tree times with TBS/0.1% Tween-20 for 10 min each and incubated with HRP-conjugated secondary antibodies diluted in TBS/0.1% Tween-20/3.5% milk for one hour, then washed with TBS/0.1% Tween-20 and finally developed using the chemiluminescent HRP substrate Immobilon Western (Millipore, Burlington, MA, USA). Primary antibodies included anti-AKNA (Santa Cruz Biotechnology, Catalogue: sc-162517) at 1:300, anti-p53 DO-1 (Santa Cruz Biotechnology, Catalogue: sc-126) at 1:1000, anti-CD40 (Abcam, Catalogue: ab65853) at 1:1000, anti α-actinin at 1:5000 (Santa Cruz Biotechnology, Catalogue: sc-17829), anti-GAPDH (Santa Cruz Biotechnology. Catalogue: sc-32233) at 1:1000, anti-AKNA (Abcam, Catalogue: ab178591) 1:250, anti-HA (Roche. Catalogue: 11 583 816 001, Basel, Switzerland) at 1:1000 and anti-HPV18 E6 (Santa Cruz Biotechnology, Catalogue: sc-365089), at 1:400. The generated anti-AKNA serum was used at 1:1000. Membranes were first incubated with the anti-AKNA antiserum, then washed and stripped to remove antibodies and finally re-probed for the other antibodies.

### 4.6. Immunofluorescence Analysis

Cells were seeded on coverslips in 6 well plates and after the indicated treatments, cells were fixed in 3.7% paraformaldehyde/PBS for 10 min and permeabilized using 0.1% Triton-X100, then cells were incubated for 2 h at 37 °C with the specified primary antibodies, washed with PBS and then incubated with anti-goat, -mouse and -rabbit Alexa fluor 488, 555 and 633 coupled secondary antibodies (Molecular Probes, Eugene, OR, USA) for 30 min at 37 °C. Slides were washed and mounted using ProLong antifade mountant with DAPI (Molecular Probes). HeLa silenced cells were processed in the same manner after 72 h postransfection and incubated with anti-p53, anti-CD40 and anti-AKNA as indicated in the figure legends. Slides were analyzed either with a fluorescence microscope EVOS FL (Thermo Ficher Scientific) or an Eclipse Ti confocal microscope with lasers (NIKON, Melville, NY, USA) giving 488, 543 and 633 nm excitation lines equipped with a NIKON camera under the 40× objective, as indicated. Collected data was processed using IMAGE-J software (NIH, Bethesda, MD, USA).

### 4.7. Fluorescence Quantification

Obtained images were analyzed using the ImageJ 1.52H software. The level of fluorescence for each condition was determined by the Corrected Total Cell Fluorescence formula (CTCF) as follows: CTCF = Integrated Density−(Area of selected cell × Mean fluorescence of background readings). Obtained CTCF values were graphed and statistical analysis performed.

### 4.8. Pull Down Assays

DH5-α *E. coli* containing the GST-E6 expressing plasmids were growth and expression induced using 1 μM IPTG for 3 h. Cell pellet was lysed with 1% Triton/PBS, sonicated for 1 minute and separated by centrifugation, supernatant cell lysate was incubated with glutathione agarose beads (Sigma Aldrich), washed and protein expression evaluated by SDS-PAGE and Coomassie staining. For pull down assays, equal amounts of purified GST fusion proteins were incubated with HaCaT cell extract for 4 h, after several washes the bound protein was separated by SDS-PAGE and analyzed by western blot.

### 4.9. Immunoprecipitation Assays

RIPA extracted protein of HeLa cells was incubated with anti-E6 (HPV18), anti-p53 and anti-AKNA antibodies (Santa Cruz Biotechnologies, Dallas, TX, USA) at a dilution of 1:50. Extracts were incubated overnight at 4 °C and then 20 μL of protein A/G Plus-agarose (Santa Cruz Biotechnologies) was added and incubated for 2 h. After three washes with RIPA buffer protein was analyzed by SDS-PAGE and western blot.

### 4.10. Proteasome Inhibition

Cells were treated with Z-Leu-Leu-Leu-Al proteasome inhibitor (Sigma Aldrich, 20 nM) for 3 h prior harvesting or fixing as specified.

### 4.11. Half-Life Assay

HaCaT cells were transfected with pCA, pCA HPV16-E6 and pCA HPV18- E6. After 24 h, cells were treated with 50 µg/mL of cycloheximide to stop protein synthesis. Cells were collected at 0, 3, 6 and 9 h post-treatment and protein extracted by adding sample loading buffer. Proteins were separated by SDS-PAGE and analyzed by immunoblot. Relative protein levels were graphed, and statistical analysis performed.

### 4.12. Anti-AKNA Antibody Generation

Antibody was generated in the laboratory by injecting the purified pcDNA3-*akna* plasmid (50 μg) in a group of 5 BALB C-21 mice; an additional group was injected with pcDNA3 and analyzed as a control. Every two weeks (from week 0 to week 6) the plasmids were inoculated in the mice. Serum was obtained and analyzed every 2 weeks (from the beginning until week 12) for AKNA detection by western blot and subsequent analyses in this study. To demonstrate that the antibodies detect AKNA, we induced the expression of AKNA from the pRSET-akna plasmid in bacteria, which include the AT-hook domain. Using serum coming from the immunized mice we detected a protein of approximately 50 KDa in bacterial lysate and cell lines. Pre-immune serum was tested to ensure the absence of unspecific signals (Supplementary Figure S2).

### 4.13. RT-PCR and qPCR

HeLa cells were transfected with 4 µg of the indicated plasmids using lipofectamine 2000 (Invitrogen). After 24 h of transfection cells were harvested and total RNA was purified using Trizol reagent (Roche, Basel, Switzerland) according to the manufacturer’s instructions. Complementary DNA was synthesized using 1 µg of total RNA by reverse transcription. To verify cDNA integrity, the human glyceraldehyde-3-phosphate dehydrogenase (GAPDH) housekeeping constitutive gene [53] was amplified by PCR. IL-8 mRNA was amplified using Fwd 5′-GGCACAAACTTTCAGAGACAG and Rev 5′-ACACAGAGCTGCAGAAATCAGG primers [54], and analyzed in an agarose gel. For qPCR Hypoxanthine Phosphoribosyltransferase 1 (HPRT1) and IL-8 mRNAs were amplified using 200 ng of cDNA and the SYBR Green Mater Mix (Applied Biosystems, Foster City, CA, USA). Data were analyzed using the equation: amount of target = 2^−ΔΔCT^, using the average ΔCT from non-treated cells as calibrator [55].

### 4.14. Flow Cytometry

Transfected cells were harvest with 1 mM EDTA solution at 37 °C, washed and suspended in Hank’s solution (HBSS). For extracellular staining cells were incubated during 30 min in dark conditions with anti-human CD40 Alexa Fluor 488 conjugated antibody (Biolegend, San Diego, CA, USA), washed with HBSS and fixed with 2% paraformaldehyde. For intracellular staining, cells were previously permeabilized with the BD intrasure kit (Becton Dickinson, Franklin Lakes, NJ, USA) according to manufacturer’s instructions. After, cells were incubated with anti CD40 antibody for 15 min and fixed as described. Fluorescence intensity was analyzed by flow cytometry in a FACS Aria II cytometer (Becton Dickinson). Analysis was performed on 10,000 events using the FlowJo 8.7 software.

### 4.15. Statistical Analysis

Experiments were performed in triplicate and data were analyzed as the mean ± SD. CD40 analysis and IL-8 mRNA expression normalized by HPRT1 (hypoxanthine phospho-ribosyl transferase) were analyzed using the student’s *t*-test in the STATA program, version 14.0 (StataCorp, Collage Station, TX, USA). Difference in protein expression was analyzed using ANOVA and Tukey’s test to determine the statistical significance of the experimental condition versus the control. For fluorescence analyses CFCT values were calculated and compared using the Student’s *t*-test comparing to the siLuc control. Differences were accepted as significant when *p* ≤ 0.05.

## 5. Conclusions

Taken together, our results demonstrate that the transcriptional factor AKNA is regulated by HPV E6 oncoprotein with the consequent deregulation of CD40, suggesting that HR-HPVs can escape from host immune surveillance by modulating pro-inflammatory responses in infected epithelial cells, resulting in persistent infections and potential carcinogenesis.

## Supplementary Materials

The following are available online at http://www.mdpi.com/2072-6694/10/12/521/s1, Figure S1: AKNA specificity control for recovery after proteasome inhibition, Figure S2: AKNA anti-serum specificity.
