# Supplementary material for: The Human Papillomavirus (HPV) E6 Oncoprotein Regulates CD40 Expression via the AT-Hook Transcription Factor AKNA
Source: Cancers (Basel). 2018 Dec 17;10(12):521. doi: 10.3390/cancers10120521 (PMC6316281; doi:10.3390/cancers10120521)
Supplement: Supplementary file 1 [file cancers-10-00521-s001.pdf]

Supplementary material

The Human Papillomavirus (HPV) E6 Oncoprotein Regulates CD40 Expression via the AT-Hook Transcription Factor AKNA

Joaquin Manzo-Merino, Alfredo Lagunas-Martínez, Carla O. Contreras-Ochoa, Marcela Lizano, Leonardo J. Castro-Muñoz, Crysele Calderón-Corona, Kirvis Torres-Poveda, Alicia Román-Gonzalez, Rogelio Hernández-Pando, Margarita Bahena-Román and Vicente Madrid-Marina

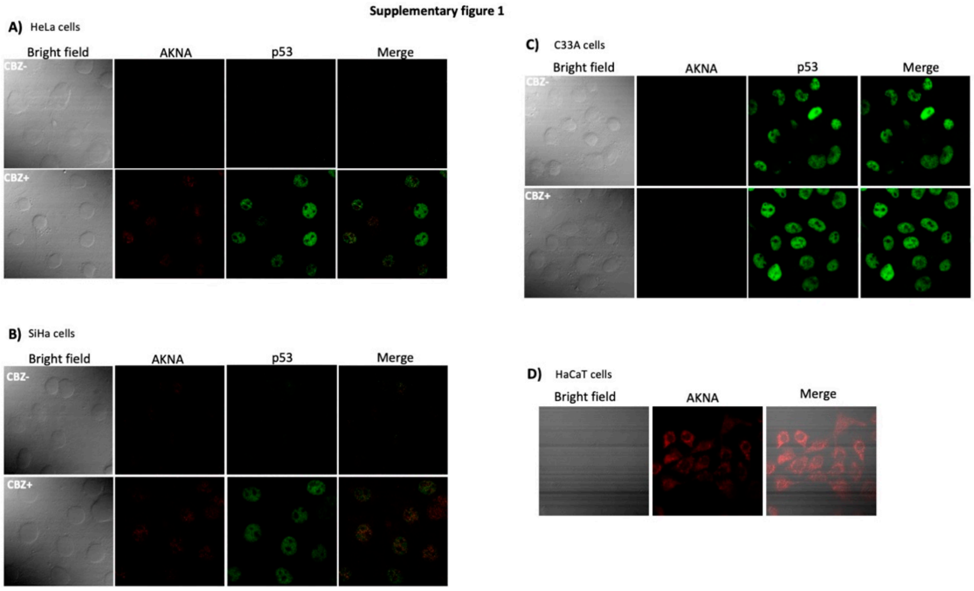

Figure S1. AKNA specificity control for recovery after proteasome inhibition. (**A**) HeLa, (**B**) SiHa and (**C**) C33A cells, were seeded on coverslips in 6 well plates and incubated to allow adherence, 24 h after seeding cells were treated either with proteasome inhibitor (CBZ) or DMSO (vehicle) for 3 h. After the incubation time, cells were fixed and stained using anti-AKNA antibody (Abcam) and p53 antibody (Santa Cruz Biotechnologies) followed by Alexa 488 (green) or Alexa 555 (red) secondary antibodies (Molecular Probes). (**D**) HaCaT cells were seeded and fixed for AKNA staining using anti-AKNA antibody (Abcam) followed by Alexa 555 (red) secondary antibody (Molecular Probes). Slides were mounted using Fluoromont media (Sigma Aldrich) and observed under the confocal NIKON Eclipse Ti microscope. HPV positive cell lines, HeLa and SiHa, exhibited a recovery in the levels of p53 in the nucleus and AKNA levels were also recovered after proteasome inhibition with a nuclear spotted pattern. C33A cells do not show AKNA recovery at all after proteasome inhibition. HaCaT cells exhibit a strong AKNA staining in the cytoplasm and the nucleus. All images were acquired under the 40× objective using a NIKON camera.

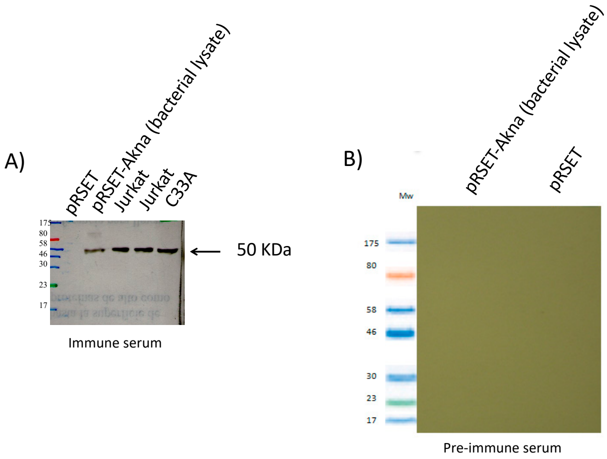

Figure S2. AKNA anti-serum specificity. Mice were immunized using pcDNA3-*akna* plasmid intramuscularly. (**A**) After 12 weeks obtained anti-serum was tested in different cell lines as well as in a protein bacterial lysate, where AKNA protein was expressed from pRSET plasmid under the T7 promoter control. (**B**) Pre-immune serum was tested in pRSET bacterial extract and pRSET-akna bacterial extract showing the absence of unspecific signals. Western blot analysis indicates the specificity of the antibody. This antibody was used in the subsequent analyses.

© 2018 by the authors. Licensee MDPI, Basel, Switzerland. This article is an open access article distributed under the terms and conditions of the Creative Commons Attribution (CC BY) license (http://creativecommons.org/licenses/by/4.0/).
